# Impact of the duration of antibiotics on clinical events in patients with *Pseudomonas aeruginosa* ventilator-associated pneumonia: study protocol for a randomized controlled study

**DOI:** 10.1186/s13063-017-1780-3

**Published:** 2017-01-23

**Authors:** Adrien Bouglé, Arnaud Foucrier, Hervé Dupont, Philippe Montravers, Alexandre Ouattara, Pierre Kalfon, Pierre Squara, Tabassome Simon, Julien Amour, for the iDIAPASON study group

**Affiliations:** 1 Department of Anesthesiology and Critical Care, CHU La Pitié-Salpêtrière, Assistance Publique – Hôpitaux de Paris (APHP), Paris, France; 2 Department of Anesthesiology and Critical Care, Hôpital Beaujon, APHP, Paris, France; 3 Department of Anesthesiology and Critical Care, CHU Amiens, Amiens, France; 4 Université de Picardie Jules Verne, Amiens, France; 5 Department of Anesthesiology and Critical Care, CHU Bichat, APHP, Paris, France; 6 Université Diderot, Paris, France; 7 Department of Anesthesiology and Critical Care, Groupe Hospitalier Sud, Pessac, Bordeaux, France; 8 Université Bordeaux Segalen, Bordeaux, France; 9 Intensive Care Unit, Hôpital Louis Pasteur, CH de Chartres, Chartres, France; 10 Intensive Care Unit, Clinique Ambroise Paré, Neuilly-sur-Seine, France; 11 Unité de Recherche Clinique du GH HUEP (URC-Est), Hôpital Saint-Antoine, APHP, Paris, France; 12 UPMC – Sorbonne universités (Paris 6), Paris, France

**Keywords:** Antibiotic, Antibiotic resistance, Bacterial infection, Bacterial pneumonia, Intensive care, Nosocomial infections, *Pseudomonas aeruginosa*, Respiratory tract infection, Ventilator-associated pneumonia

## Abstract

**Background:**

Ventilator-associated pneumonia (VAP) accounts for 25% of infections in intensive care units. Compared to a long duration (LD) of antibiotic therapy, a short duration (SD) has a comparable clinical efficacy with less antibiotic use and less multidrug-resistant (MDR) pathogen emergence, with the exception of documented VAP of non-fermenting Gram-negative bacilli (NF-GNB), including *Pseudomonas aeruginosa* (PA). These results have led the American Thoracic Society to recommend SD therapy for VAP, except for PA-VAP. Thus the beneficial effect of SD therapy in PA-VAP is still a matter of debate. We aimed to assess the non-inferiority of a short duration of antibiotics (8 days) versus prolonged antibiotic therapy (15 days) in PA-VAP.

**Methods/design:**

The impact of the duration of antibiotics on clinical events in patients with *Pseudomonas aeruginosa* ventilator-associated pneumonia (iDIAPASON) trial is a randomized, open-labeled non-inferiority controlled trial, conducted in 34 French intensive care units (ICUs), comparing two groups of patients with PA-VAP according to the duration (8 days or 15 days) of effective antibiotic therapy against PA. The primary outcome is a composite endpoint combining day 90 mortality and PA-VAP recurrence rate during hospitalization in the ICU. Furthermore, durations of mechanical ventilation and hospitalization, as well as number and types of extrapulmonary infections or acquisition of MDR pathogens during the hospitalization in the ICU will be recorded. Recurrence with predefined criteria (clinical suspicion of VAP associated with a positive quantitative culture of a respiratory sample) will be evaluated by two independent experts.

**Discussion:**

Demonstrating that an SD (8 days) versus LD (15 days) therapy strategy in PA-VAP treatment is safe and not associated with an increased mortality or recurrence rate could lead to a change in practices and guidelines in the management of antibiotic therapy of this frequent ICU complication. This strategy could lead to decreased antibiotic exposure during hospitalization in the ICU and in turn reduce the acquisition and the spread of MDR pathogens.

**Trial registration:**

ClinicalTrials.gov: NCT02634411. Registered on 19 November 2015.

**Electronic supplementary material:**

The online version of this article (doi:10.1186/s13063-017-1780-3) contains supplementary material, which is available to authorized users.

## Background

Ventilator-associated pneumonia (VAP) is a major cause of morbidity and mortality in intensive care units (ICUs), accounting for 25% of infections in these units [1]. From 1975 to 2003, the incidence of hospital-acquired pneumonia caused by *Pseudomonas aeruginosa* (PA) has almost doubled, increasing from 9.6% to 18.1% [2], and PA is the most frequently isolated Gram-negative aerobic bacterium from ICUs in a US national large-scale survey (23%) and the most frequent bacterium isolated from the respiratory tract (32%) [3]. PA-VAP is associated with a high mortality ranging from 40% up to 69% [4–7] and with rates of recurrence more than 30% despite adequate antimicrobial therapy [7–9].

More than 60% of all ICU patients receive antibiotics during their hospitalization [10]. It is critical to reduce antibiotic exposure of ICU patients without compromising patient safety, and in particular without more recurrences. Hence, few studies have reported the recurrence rate according to the antibiotic therapy duration. In a randomized non-inferiority trial comparing 8 days versus 15 days of antibiotic duration strategies for VAP, the mortality and recurrence rate 28 days after bronchoscopy did not significantly differ between groups [9]. However, patients with VAP caused by non-fermenting Gram-negative bacilli (NF-GNB), including PA, had a higher rate of recurrence with short-duration (SD) therapy compared to a long-duration (LD) therapy group [9]. These results have led the American Thoracic Society to recommend SD therapy for VAP, with the exception of VAP with NF-GNB, including *Pseudomonas aeruginosa* (PA-VAP) [11]. Nevertheless, many trials have shown that decreased antibiotic duration for PA-VAP would be safe and not associated with more recurrences or increased mortality [12, 13]. Hence, in a multicentric, randomized, controlled trial (the ProVAP study), an antibiotics discontinuation strategy according to serum procalcitonin concentrations allowed antibiotic exposure to be reduced without increasing recurrences or mortality, even though PA was the most frequently isolated bacterium [14]. Recently, duration of adequate antibiotic therapy was found to be not associated with treatment failure in a retrospective cohort of patients with PA-VAP [7]. Moreover, three trials [15–17] and a Cochrane meta-analysis [18] failed to demonstrate that a short fixed-course (7 or 8 days) antibiotic therapy could be associated with an increase in mortality or recurrence rate for patients with a pneumonia documented with PA or other NF-GNB. Finally, a recent meta-analysis concluded that the two strategies did not differ in terms of 28 day mortality, but because of lack of power, these conclusions remain a matter of debate [19].

Thus, whereas SD therapy strategy has shown its efficacy and safety in patients with VAP non-caused by NF-GNB, allowing decreasing antibiotic exposure without excess morbidity or mortality, its use in patients with documented VAP of NF-GNB remains a matter of debate. The impact of the duration of antibiotics on clinical events in patients with *Pseudomonas aeruginosa* ventilator-associated pneumonia (iDIAPASON) study was thus designed to evaluate this important clinical issue.

## Methods/design

The study is a randomized, open-labeled non-inferiority trial comparing two parallel groups: those receiving 8 days of antibiotic therapy (short duration, SD) and those receiving 15 days of antibiotic therapy (long duration, LD).

### Study population

Patients aged ≥18 years, diagnosed with ventilator-associated pneumonia documented with *Pseudomonas aeruginosa* (PA-VAP), affiliated to French social security, and providing a written informed consent signed by themselves or their next of kin are eligible for inclusion. The diagnosis of PA-VAP will include a clinical suspicion (≥ two criteria including fever > 38.5 °C, leukocytosis > 10^9^/L or leukopenia < 4.10^8^/L, purulent tracheobronchial secretions, and a new or persistent infiltrate on chest radiography) and confirmation by a *Pseudomonas aeruginosa* positive quantitative culture of a respiratory sample: bronchoalveolar lavage fluid (significant threshold ≥10^4^ colony-forming units (CFU)/mL) or plugged telescopic catheter (significant threshold ≥10^3^ CFU/mL) or quantitative endotracheal aspirate distal pulmonary secretion samples (significant threshold ≥10^6^ CFU/mL) [20].

Patients are not eligible in case of the following conditions: pregnancy, immunosuppression (HIV, immunosuppressive therapy, corticosteroids >0.5 mg/kg per day for more than a month), current antibiotic therapy active on PA for extrapulmonary infection, procedure of withdrawing life-sustaining treatment, chronic pulmonary colonization with *Pseudomonas aeruginosa* (chronic obstructive pulmonary disease (COPD) or bronchiectasis, with a positive respiratory sample below the threshold rate for *Pseudomonas aeruginosa* (i.e., <10^3^ CFU/mL for protected specimen brush or <10^6^ CFU/mL for tracheal aspirate), obtained in the absence of pneumonia or exacerbation during the 6 months before the ICU admission).

**Fig. 1:**
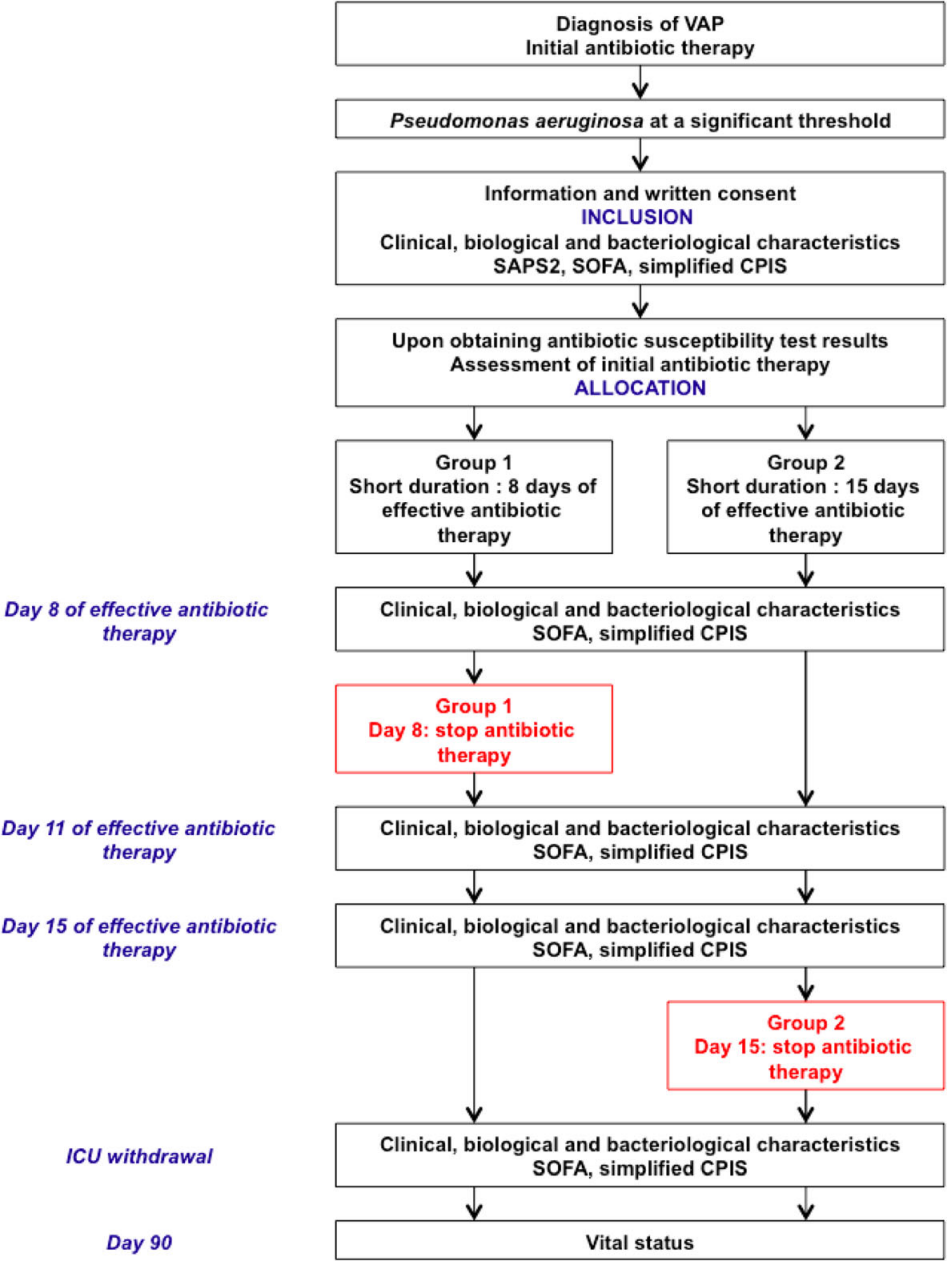
Conduct of the study. Study protocol flowchart. *SAPS* 2: Simplified acute physiology score II; *SOFA:* Sequential organ failure assessment; *CPIS:* clinical pulmonary infection score

**Fig. 2:**
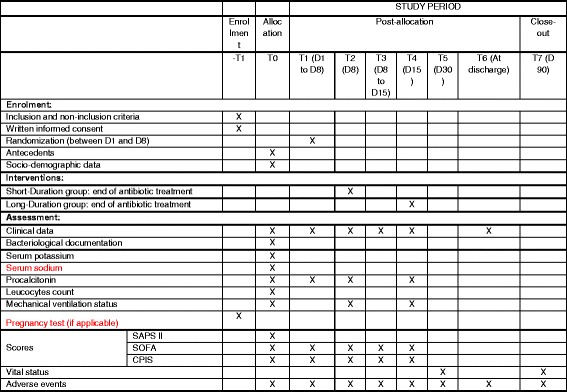
Template of recommended content for the schedule of enrollment, interventions, and assessment. *SAPS 2*: simplified acute physiology score (SAPS II); *SOFA*: sequential organ failure assessment (SOFA) score; *CPIS*: clinical pulmonary infection score

Antibiotic treatment should be started just after realization of bacteriological sampling, without waiting for the result. The choice of initial antibiotic therapy will be left to the discretion of the physician according to usual care based on the clinical context, previous antibiotic therapy, the presence or absence of risk factors for multidrug-resistant (MDR) pathogen antibiotics or hospitalization in the previous 90 days (current hospitalization ≥5 days, mechanical ventilation ≥5 days, support in a dialysis center or residency in a nursing home), local epidemiological data, and finally knowledge that the patient is already known as being colonized by a MDR pathogen. In these situations, a broad-spectrum antibiotic will be recommended immediately, with the association of a β-lactam/β-lactamase inhibitor or an antipseudomonal cephalosporin, and an aminoglycoside or an antipseudomonal fluoroquinolone for 3 to 5 days. An initial antibiotic with a narrow spectrum will be accepted in the face of early onset pneumonia (mechanical ventilation <5 days) and in the absence of risk factors for MDR pathogens and in accordance to the marketing authorization (AMM), contraindications, dosing recommendations, and drug interactions (http://base-donnees-publique.medicaments.gouv.fr/). Investigators are strongly encouraged to convert this initial regimen into a narrow-spectrum therapy, based on culture results. All antibiotics would be withdrawn, either at the end of day 8 or day 15, according to the randomization assignment, except those prescribed for a documented pulmonary infection recurrence or a new extrapulmonary infection before that day. The patient will be randomized just after antibiotic susceptibility test results and assigned to the SD arm or the LD arm. Screening of MDR pathogens will be realized with a surveillance culture of swab samples from the rectum for extended-spectrum β-lactam-producing Enterobacteriaceae (ESBL) and of swab samples from the anterior nares for methicillin-resistant *Staphylococcus aureus* (MRSA) at the patient’s admission in the ICU, and then once weekly until the patient’s discharge from the ICU (see Figs. 1 and 2).

### Partners

Dr. Adrien Bouglé, coordinating investigator, Hôpital Universitaire La Pitié-Salpêtrière (France), is responsible for the study. Pr. Julien Amour is the scientific director, and the methodological support will be provided by the Clinical Research Unit Est (Pr. Tabassome Simon, Hôpital Saint Antoine, APHP, Paris, France). A steering committee includes the coordinating investigator, the scientific director, nine French ICU experts, and the head of the Clinical Research Unit (see Table 1 for the composition and affiliations of the members of the steering committee). This committee will control the proper conduct of the trial in terms of methodological, ethical, and logistical aspects.

**Table 1:** Investigators and centers

Investigators	Centers
Coordinating Investigator	
BOUGLÉ Adrien	CHU La Pitié-Salpêtrière, Paris
Scientific Director	
AMOUR Julien	CHU La Pitié-Salpêtrière, Paris
Co-investigators	
BOUGLÉ Adrien	CHU La Pitié-Salpêtrière, Paris
MAYAUX Julien	CHU La Pitié-Salpêtrière, Paris
LESCOT Thomas	CHU Saint Antoine, Paris
LANGERON Olivier	CHU La Pitié-Salpêtrière, Paris
POETE Pascale	CHU La Pitié-Salpêtrière, Paris
OUATTARA Alexandre	CHU Bordeaux
BELLENFANT ZEGDI Florence	Hôpital Européen Georges Pompidou, Paris
CHOLLEY Bernard	Hôpital Européen Georges Pompidou, Paris
PAUGAM Catherine	CHU Beaujon, Paris
MONTRAVERS Philippe	CHU Bichat, Paris
DURANTEAU Jacques	CHU Bicêtre, Paris
COUSSON Joël	CHU Reims
DUPONT Hervé	CHU Amiens
GARNIER Marc	Hôpital Tenon, Paris
LEONE Marc	Hôpital Nord, Marseille
GEERAERTS Thomas	CHU Toulouse
LEFRANT Jean-Yves	CHU Nîmes
VEBER Benoit	CHU Rouen
GUICHON Céline	Hôpital Croix Rousse, Lyon
SQUARA Pierre	Clinique Ambroise Paré, Neuilly-sur-Seine
LASOCKI Sigismond	CHU Angers
MIRA Jean-Paul	Hôpital Cochin, Paris
BAUDEL Jean-Luc	Hôpital Saint-Antoine, Paris
PAYEN Jean-François	CHU Grenoble
KIPNIS Eric	CHRU Lille
POTTECHER Julien	CHRU Strasbourg
LEGRAND Matthieu	Groupe Hospitalier Lariboisière – Saint Louis, Paris
TESNIERE Antoine	Hôpital Cochin, Paris
CONSTANTIN Jean-Michel	CHU Clermont-Ferrand
PERTEK Jean-Pierre	CHU Nancy Brabois
PICARD Walter	CH Pau
FEDERICI Laura	CH Sud Francilien, Corbeil-Essonnes
LEVY Bruno	CHU Nancy Brabois
RABBAT Antoine	Hôpital Cochin, Paris
LEVESQUE Eric	Hôpital Henri Mondor, Créteil
MONGARDON Nicolas	Hôpital Henri Mondor, Créteil
CONIA Alexandre	CH Chartres
HISSEM Tarik	CH Sud Essonne, Étampes
FAURE Henri	CHI Robert Ballanger, Aulnay-Sous-Bois
Steering Committee	
BOUGLÉ Adrien (chair)	CHU La Pitié-Salpêtrière, Paris
AMOUR Julien	CHU La Pitié-Salpêtrière, Paris
SIMON Tabassome	URC-Est, Paris
KALFON Pierre	CH Chartres
FOUCRIER Arnaud	CHU Beaujon, Paris
DUPONT Hervé	CHU Amiens
CHOLLEY Bernard	Hôpital Européen Georges Pompidou, Paris
CONSTANTIN Jean-Michel	CHU Clermont-Ferrand
LEONE Marc	Hôpital Nord, Marseille
MONTRAVERS Philippe	CHU Bichat, Paris
OUATTARA Alexandre	CHU Bordeaux
SQUARA Pierre	Clinique Ambroise Paré, Neuilly-sur-Seine
VEBER Benoit	CHU Rouen

The study will be performed in 39 French adult ICUs (Table 1). This work is supported by institutional grants from the French 2014 Programme Hospitalier de Recherche Clinique National (PHRC P140923 – AOM14515). The study is sponsored by the Clinical Research and Development Department of Assistance Publique – Hôpitaux de Paris. The sponsor had no role in the study design, collection, management, analysis, or interpretation of data, writing of the report, or the decision to submit the report for publication. The total duration of the study will be 27 months, including the inclusion period (24 months) and the duration of participation per patient (90 days).

### Objectives

The primary objective will be to assess the non-inferiority of a short duration of antibiotics (8 days) compared to prolonged antibiotic therapy (15 days) in *Pseudomonas aeruginosa* ventilator-associated pneumonia (PA-VAP) on morbidity-mortality at 90 days.

The secondary objectives will be to compare between short and long durations of antibiotics regarding:

- Mortality in the ICU
- Morbidity in the ICU (mechanical ventilation, duration of hospitalization)
- Exposure and acquisition of MDR pathogens during hospitalization
- Number and types of extrapulmonary infections

### Assessment criteria

The primary assessment criterion will be a composite endpoint combining day 90 mortality and PA-VAP recurrence rate during hospitalization in the ICU (within 90 days). Recurrence will be defined with a post hoc analysis by two independent experts with predefined criteria: clinical suspicion of VAP (≥ two criteria including: fever >38.5 °C, leukocytosis >10^9^/L or leukopenia <4.10^8^/L, purulent tracheobronchial secretions, and a new or persistent infiltrate on chest radiography) associated with a positive quantitative culture of a respiratory sample (bronchoalveolar lavage fluid (significant threshold ≥10^4^ CFU/mL), plugged telescopic catheter (significant threshold ≥10^3^ CFU/mL), or quantitative endotracheal aspirate distal pulmonary secretion samples (significant threshold ≥10^6^ CFU/mL)). In cases of disagreement between the two experts, a third expert will reach a consensus. Thereby, the percentage of patients with PA-VAP recurrence and/or death will be assessed.

Secondary assessment criteria will be 30 day and 90 day mortality rate, PA-VAP recurrence rate, mechanical ventilation (MV)-free days, ICU-free days, antibiotic-free days during the hospitalization in the ICU, number and types of extrapulmonary infections during the hospitalization in the ICU, and acquisition of MDR pathogens during the hospitalization in the ICU (from swab sample of rectum and anterior nares).

### Data collection

All efficacy parameters will be collected during hospitalization of the patient in the ICU. In case of hospital discharge, phone calls will be made by the investigators or the research team with at least three attempts to contact the patient, his/her physician, or the patient’s emergency contact in a period of 10 days at different times of the day. If there is no answer, vital statistics will be collected via contact of the town council of the patient’s birthplace.

Data will be collected by the investigators and their teams in an electronic case report form (e-CRF). Data will be monitored by a clinical research associate (CRA) and checked for consistency and missing values by a data manager.

### Randomization

Centralized blocked-balanced randomization, in a 1:1 ratio, will be computer-generated by the clinical research unit (URC-EST). Randomization will be stratified on center. Investigators are blinded to the block size.

### Statistical analysis

The study is designed to demonstrate the non-inferiority on the composite endpoint of mortality and recurrence at 90 days of the 8-day strategy versus the 15-day strategy for PA-VAP. Considering the mortality and/or recurrence of PA-VAP rate of 35.7% [7], 284 patients will be required in each group to achieve a power of 80% to exclude a 10% difference between the two groups with an α risk of 5%. To account for a possible 5% patient loss to follow-up, we planned to enroll 600 patients.

### Data analysis

Baseline characteristics of patients will be described per group. Qualitative data will be described with frequencies and percentages; quantitative data will be described with mean and standard error or with median, interquartile interval, and range. Since this is a non-inferiority study, analysis of principal criteria will be performed on a per protocol population. The per protocol population will be defined as randomized patients without major protocol deviations such as non-respect of all selection criteria, clear non-respect of the antibiotic duration allocated (+/– 24 hours), missing data for the primary endpoint, and major protocol deviation(s) identified during a blinded data review before data base freezing. Non-inferiority will be tested by a Dunnett and Gent chi-squared procedure [21]. Secondary analysis in an intent-to-treat (ITT) population will also be performed.

Secondary outcomes will be analyzed under a superiority assumption. Day 30 and day 90 mortality rates and acquisition of MDR pathogens during hospitalization in the ICU will also be compared between groups by a chi-squared test or a Fisher’s exact test, if the use conditions were not verified. Duration of mechanical ventilation, duration of hospitalization in the ICU, duration of exposure to antibiotics, and number of extrapulmonary infections during hospitalization in the ICU will be compared between groups by Student *t* tests or Mann-Whitney/Wilcoxon tests, if needed. Types of extrapulmonary infections will be described.

The association between respiratory colonization with yeast at diagnosis and PA recurrence will be studied by logistic regression adjusted on the randomization group. The association between monotherapy or combination therapy and mortality and PA recurrence will be studied by a logistic regression adjusted on the randomization group. Procalcitonin levels at days 0, 1, 3, and 7 will be described, and their prognosis values on mortality and *P. aeruginosa* recurrence will be studied by logistic regression and sensitivity and specificity analyses. Missing data will be not replaced.

The study will be conducted in accordance with the Helsinki Declaration and French laws and regulations. The study has received its approval from the French ethics committee (Comité de Protection des Personnes Ile de France VI) as well from the French Drug Safety Agency (Agence Nationale de Sécurité du Médicament et des Produits de Santé) (EudraCT 2015-003102-17).

All records and subjects’ identities will remain confidential in accordance with the following French regulations: the French National Committee of Informatics and Liberties and the French Consultative Committee for Data Processing in Health Research rules. The trial protocol was written following the Recommendations for Interventional trials (SPIRIT) Checklist (see Additional file 1).

## Discussion

This study will investigate the influence of duration of antibiotic therapy on mortality and recurrence of PA-VAP. Our goal is to demonstrate that a short antibiotic therapy (8 days of effective antibiotic therapy) compared to a long antibiotic therapy (15 days of effective antibiotic therapy) is safe without excess mortality or recurrence, and decreases the duration of exposure to antibiotics and the acquisition of MDR pathogens. Furthermore, this study will evaluate the relationship between PA recurrence and (1) respiratory colonization with yeast at diagnosis, (2) treatment with monotherapy or combination therapy, and (3) procalcitonin levels during the course of VAP.

We believe that the present study has several strengths. First, it specifically addresses the issue of recurrence of PA-VAP, and the number of patients to be included (*n* = 600) has been calculated according to an expected rate of 35% for the composite endpoint combining 90 day mortality and PA-VAP recurrence as shown in a previous study [7]. This expected rate is in accordance with previously published data. Hence, Combes et al. reported a recurrence rate of 28% [22]. In a meta-analysis, the frequency of VAP recurrence was 26.8%. Acute lung injury/acute respiratory distress syndrome and shock on the day of diagnosis of the first VAP episode, but not the first-episode causative pathogens, were found to be associated with VAP recurrence [23]. Finally, in the cohort published by Planquette et al. on 314 patients with 393 PA-VAP, failure occurred for 112 of them (35.7%), including 79 recurrences (20.1%) [7].

Crouch Brewer et al. reported in a cohort of 38 consecutive PA-VAP a mortality of 69% with an attributable mortality of 38% [4]. We can emphasize that persistent pneumonia occurred in 35% of patients while recurrence of pneumonia was unusual (1/38). Trouillet at al. reported a cohort of 135 PA-VAP. In this cohort, 70 patients (52%) died during the ICU stay, and only 10 patients had recurrences [5]. Finally, in a retrospective, multicenter, observational cohort study conducted in five Spanish ICUs, Garnacho-Montero et al. reported 183 monomicrobial episodes of PA-VAP, with a mortality of 42.1% [6], and an Italian retrospective analysis of data prospectively collected about 110 cases of PA-VAP and reported an ICU mortality of 44.5% [24].

The study is open-labeled, because a double-blind study would have presented considerable financial and organizational constraints. Thus, we cannot completely rule out the risk of bias, especially the non-compliance with the antibiotic therapy duration. Nevertheless, first, investigators received clear instructions about the crucial importance of compliance with the treatment allocation. Second, the comparison of the duration of exposure to antibiotics between groups and between centers will give us an indication about non-compliance with study arms. Third, post hoc analysis of the primary endpoint will be conducted without any knowledge of the study arm.

In conclusion, this study should contribute to determine the most appropriate duration of antibiotic therapy treatment in the specific case of PA-VAP to decrease both mortality and the recurrence rate of PA-VAP. If our hypothesis is confirmed, the study may lead to decreased antibiotic exposure during the hospitalization in ICU and in turn reduce the acquisition and the spread of MDR. The therapeutic perspectives which could result from this study are thus promising.

### Trial status

The first patients were randomized in June 2016. The inclusion of participants is ongoing and is expected to continue until June 2018.

## Additional file

Additional file 1: SPIRIT checklist. (DOC 147 kb)

## Abbreviations

COPD: Chronic obstructive pulmonary disease
ICU: Intensive care unit
MDR: Multidrug-resistant (pathogens)
PA: *Pseudomonas aeruginosa*
PCT: Procalcitonin
VAP: Ventilator-associated pneumonia
