# Supplementary material for: Impact of the duration of antibiotics on clinical events in patients with Pseudomonas aeruginosa ventilator-associated pneumonia: study protocol for a randomized controlled study
Source: Trials. 2017 Jan 23;18:37. doi: 10.1186/s13063-017-1780-3 (PMC5260072; doi:10.1186/s13063-017-1780-3)
Supplement: Additional file 1: — SPIRIT checklist. (DOC 147 kb) [file 13063_2017_1780_MOESM1_ESM.doc]

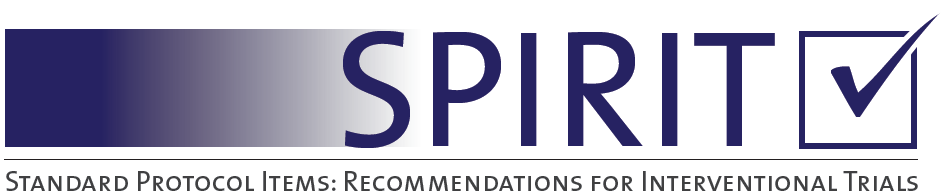


SPIRIT 2013 Checklist: Recommended items to address in a clinical trial protocol and related documents*

| Section/item | ItemNo | Description |
| --- | --- | --- |
| **Administrative information** | | |
| Title | 1 | Impact of the Duration of Antibiotics on clinical events in patients with PSeudomONas aeruginosa ventilator-associated pneumonia: study protocol for a randomized controlled study |
| Trial registration | 2a | This trial is registered with ClinicalTrials.gov (www.clinicaltrials.gov) on 19 November 2015, Identifier: NCT02634411. |
|  | 2b | Not applicable |
| Protocol version | 3 | Version N° 4.0 of 23/11/2016 |
| Funding | 4 | This work is supported by institutional grants from the French 2014 Programme Hospitalier de Recherche Clinique National (PHRC P140923 –AOM14515). |
| Roles and responsibilities | 5a | Coordinating Investigator: Adrien BOUGLE, MD  Anesthesiology and Critical Care Medicine  Institut de Cardiologie, Groupe Hospitalier Pitié-Salpêtrière  Assistance Publique – Hôpitaux de Paris  Tel.: +33(1) 42 16 29 91 / Email: adrien.bougle@psl.aphp.fr  Scientific Director: Julien AMOUR, MD, PhD  Anesthesiology and Critical Care Medicine  Institut de Cardiologie, Groupe Hospitalier Pitié-Salpêtrière  Assistance Publique – Hôpitaux de Paris  Tel.: +33(1) 42 16 56 41 / Email: julien.amour@psl.aphp.fr |
|  | 5b | Christine Lanau  AP-HP and by delegation: Clinical Research and Development Department (DRCD)  Hôpital Saint-Louis  1, avenue Claude Vellefaux  75010 PARIS  DRCD-Siège project referent: Christine LANAU  Tel. 01 44 84 17 89  Email: christine.lanau@drc.aphp.fr |
|  | 5c | No role of study sponsor and funders in study design; collection, management, analysis, and interpretation of data; writing of the report; and the decision to submit the report for publication |
|  | 5d | Steering committee: Dr. Adrien Bouglé (chair), Pr. Julien Amour  Pr. Tabassome Simon, Dr. Pierre Kalfon, Dr. Arnaud Foucrier, Pr. Hervé Dupont, Pr. Bernard Cholley, Pr. Jean-Michel Constantin, Pr. Marc Leone, Pr. Philippe Montavers, Pr. Alexandre Ouattara, Dr. Pierre Squara, Pr. Benoit Veber |
| Introduction |  |  |
| Background and rationale | 6a | Ventilator-associated pneumonia (VAP) accounts for 25% of infections in intensive care units (Réseau RAISIN 2012). A short duration (8 days; SD) vs. long duration (15 days; LD) of antibiotic therapy has a comparable clinical efficacy with less antibiotic use and less multidrug-resistant pathogens (MDR) emergence. These results have led the American Thoracic Society to recommend SD therapy for VAP, with the exception of documented VAP of non-fermenting Gram negative bacilli (NF-GNB), including Pseudomonas aeruginosa (PA-VAP), due to the absence of studies focusing specifically on PA-VAP. Thus the beneficial effect of SD therapy in PA-VAP is still a matter of debate. In a small (n=127) subgroup analysis, a higher rate of recurrence with SD therapy (n=21, 32.8%) has been observed compared with LD therapy group (n=12, 19.0%). Unfortunately, the definition of recurrence was essentially based on microbiological rather than clinical data, and the higher rate of recurrence observed could rather reflect a higher rate of colonization more than a new infection.  Interestingly, a trend for a lower rate of mortality was also observed in the SD group (n=15, 23.4%) compared with the LD group (n=19, 30.2%), but this study was clearly underpowered to detect a difference of mortality between groups.  The two strategies were considered as not different, for the risk of mortality in a recent meta-analysis, performed on the very few available studies (n=2), that (OR = 1.33, 95% CI [0.33 to 5.26] for SD vs. LD strategies respectively). However, this conclusion remains questionable considering the large confidence interval of the risk and the power of these studies. |
|  | 6b | Explanation for choice of comparators |
| Objectives | 7 | Primary objective  To assess the non-inferiority of a short duration of antibiotics (8 days) vs. prolonged antibiotic therapy (15 days) in Pseudomonas aeruginosa ventilator-associated pneumonia (PA-VAP) on morbi-mortality at 90 days.  Secondary objectives  To compare between short and long duration of antibiotics on:  - mortality in the ICU  - morbidity in the ICU (mechanical ventilation, duration of hospitalization)  - exposure and acquisition of MDR during hospitalization  - number and types of extrapulmonary infections |
| Trial design | 8 | Randomized, open-labeled non inferiority trial comparing to parallel groups:  - 8 days of antibiotic therapy  - 15 days of antibiotic therapy |
| Methods: Participants, interventions, and outcomes | | |
| Study setting | 9 | 39 French Intensive Care Units |
| Eligibility criteria | 10 | ***Inclusion criteria***  • Patients ≥ 18 years  • Mechanical ventilation ≥ 48 hours  • Diagnosis of PA-VAP:  - Clinical suspicion (≥ two criteria including fever> 38.5 ° C, leukocytosis > 109/L or leukopenia < 4.108/L, purulent tracheobronchial secretions and a new or persistent infiltrate on chest radiography)  - And confirmation by a Pseudomonas aeruginosa positive quantitative culture of a respiratory sample: bronchoalveolar lavage fluid (significant threshold ≥104 colony-forming units/mL) or plugged telescopic catheter (significant threshold ≥103 colony-forming units/mL) or quantitative endotracheal aspirate distal pulmonary secretion samples (significant threshold ≥106 colony-forming units/mL)  • Written informed consent signed by the patient or their next-of-kin  • Patients affiliated to French social security  ***Non-inclusion criteria***  • Pregnancy  • Patients included in another interventional study  • Immunocompromised patients (HIV, immunosuppressive therapy, corticosteroids> 0.5 mg/kg per day for more than a month)  • Patients receiving antibiotic therapy for extrapulmonary infection  • Patients in whom a procedure of withdrawing life-sustaining treatment has been established  • Chronic pulmonary colonization with Pseudomonas aeruginosa: patients with Chronic Obstructive Pulmonary Disease (COPD) or bronchiectasis, with a positive respiratory sample at infra threshold rate for Pseudomonas aeruginosa (i.e. <103 colony-forming units (CFUs)/mL for protected specimen brush or <106 CFUs/mL for tracheal aspirate), obtained in the absence of pneumonia or exacerbation during the 6 months before the ICU admission.  • Patient under guardianship or curatorship |
| Interventions | 11a | Antibiotic treatment should be started just after realization of bacteriological sampling, without waiting for the result. The choice of initial antibiotic therapy will be left to the discretion of the physician according to usual care based on the clinical context, previously antibiotic therapy, the presence or absence of risk factors for multidrug-resistant (MDR) pathogen. Investigators would be strongly encouraged to convert this initial regimen into a narrow-spectrum therapy, based on culture results. All antibiotics would be withdrawn, either at the end of day 8 (Short Duration, SD) or day 15 (Long Duration, LD), according to the randomization assignment, except those prescribed for a documented pulmonary infection recurrence or a new extrapulmonary infection before that day. The patient will be randomized just after antibiotic susceptibility test results and assigned to the SD arm or the LD arm. Screening of MDR pathogens will be realized with surveillance culture of swab samples from rectum for extended-spectrum β-lactam-producing Enterobacteriaceae (ESBL) and of swab samples from the anterior nares for Methicillin-Resistant *Staphylococcus aureus* (MRSA) at the patient’s admission in the Intensive Care Unit, and then once weekly until patient’s discharge of the ICU. |
|  | 11b | - Any subject can withdraw from participating in the research at any time and for any reason.  - The investigator can temporarily or permanently end a subject's participation in the research for any reason that affects the subject's safety or which would be in the subject's best interests.  - No medical conditions would require exclusion or withdrawal.  If a subject leaves the research prematurely, data relating to the subject can be used unless an objection was recorded when the subject signed the consent form.  If consent is withdrawn, no data about the subject may be used unless the subject states in writing that he/she does not object. In practice, the subject is excluded from the research. |
|  | 11c | No strategies implemented. |
|  | 11d | Not applicable |
| Outcomes | 12 | Primary assessment criterion  A composite endpoint combining Day-90 mortality and PA-VAP recurrence rate during hospitalization in the ICU (within 90 days).  Recurrence will be defined a posteriori by 2 independent experts with predefined criteria: clinical suspicion of VAP (≥ two criteria including: fever> 38.5 ° C, leukocytosis > 109/L or leukopenia < 4.108/L, purulent tracheobronchial secretions and a new or persistent infiltrate on chest radiography) associated with a positive quantitative culture of a respiratory sample (bronchoalveolar lavage fluid (significant threshold ≥104 colony-forming units/mL) or plugged telescopic catheter (significant threshold ≥103 colony-forming units/mL) or quantitative endotracheal aspirate distal pulmonary secretion samples (significant threshold ≥106 colony-forming units/mL)). In cases of disagreement between the two experts, a third expert will reach a consensus. Thereby, the percentage of patients with PA-VAP recurrence and/or death will be assessed.  Secondary assessment criteria  1) D30 and D90 mortality rate (%)  2) Morbidity by:  Duration of mechanical ventilation (days)  PA-VAP recurrence rate,  Duration of hospitalization in ICU (days)  3) Exposure to antibiotics during the hospitalization in the ICU (days)  4) Number and types of extrapulmonary infections during the hospitalization in the ICU (n)  5) Acquisition of MDR during the hospitalization in the ICU (swab sample of rectum and anterior nares) |
| Participant timeline | 13 | Inclusion visit – D0 The patient will be randomized just after antibiotic susceptibility test results. Screening of MDR pathogens will be realized with surveillance culture of swab samples from rectum for extended-spectrum β-lactam-producing Enterobacteriaceae (ESBL) and of swab samples from the anterior nares for Methicillin-Resistant *Staphylococcus aureus* (MRSA) at the patient’s admission in the Intensive Care Unit, and then once weekly until patient’s discharge of the ICU.  - Clinical assessment including pregnancy test (if applicable)  - Colonization with multidrug-resistant pathogens (Multidrug-resistant bacteria were defined as one of the following: ticarcillin-resistant *Pseudomonas aeruginosa, Acinetobacter baumannii*, or *Stenotrophomonas maltophilia*; extended-spectrum β-lactam-producing Enterobacteriaceae; high-concentration cephalosporinase-producing AmpC Enterobacteriaceae; and meticillin-resistant *Staphylococcus aureus*)  - Scores: SAPSII (gravity assessment at the admission) (Annexe 1); SOFA (organ failure assessment) (Annexe 1); CPIS (clinical suspicion of ventilator-associated pneumonia) (Annexe 1)   - Initiation of antibiotic therapy according to guidelines.  Follow-up VisitsAs done in usual care, the adequacy of the empirical antibiotic treatment will be controlled between D3 and D5 (results of the in vitro activity against identified Pseudomonas aeruginosa). The antibiotic will be modified if necessary. The physician, as done usually, will determinate the first day of effective antibiotic treatment. The patient will be randomized at this moment.  ***D4 of effective antibiotic treatment***  - Clinical assessment  - Laboratory assessment  - Mechanical ventilation status  - SOFA and CPIS score  ***D8 of effective antibiotic treatment***  - Clinical assessment by local physician to ensure the absence of PA-VAP recurrence  - Laboratory assessment  - Mechanical ventilation status  - SOFA and CPIS score   - End of antibiotic therapy for group 1 (Group SD: Stop antibiotics after 8 days of effective treatment).   ***D11 of effective antibiotic treatment***  - SOFA and CPIS score  - Laboratory assessment  ***D15 of effective antibiotic treatment***  - Clinical assessment  - Laboratory assessment  - Colonization with multidrug-resistant pathogens  - Mechanical ventilation status  - SOFA and CPIS scores   - End of antibiotic therapy for group 2 (Group LD: Stop antibiotics after 15 days of effective treatment). |

| Participant timeline | 13 | D30 of effective antibiotic treatment - Vital status assessment  ***At ICU discharge***  - Clinical assessment  - Mechanical ventilation status  - Antibiotic use daily throughout study period  - Any infection throughout study period: infection site, bacteriological documentation, antibiotic therapy  - Colonization with multidrug-resistant pathogens  - Patient’s status at discharge from the ICU  ***D90 of effective antibiotic treatment***  - Vital status assessment:  If the patient is no longer hospitalized, he will be called at 3 months (D90 ± 15D) for vital status assessment. The study coordinator (TEC) should make at least 3 attempts to contact the patient, his physician or the patient’s emergency contact in a period of 10 days at different time of the day. In the absence of answer, vital status will be collected via contact of the town council of the patient birthplace. |
| --- | --- | --- |

| Sample size | 14 | In a recently published study, mortality and/or recurrence of PA-VAP was 35.7%. The study is designed to demonstrate the non-inferiority on the composite endpoint of mortality and recurrence on day-90 of the 8-days strategy vs. the 15-days strategy for PA-VAP. To test the non-inferiority with a α risk of 5%, 284 patients will be required in each group to achieve a power of 80% to exclude a 10% difference between the two groups. To account for possible 5% patients lost to follow- up, we planned to enrol 600 patients. |
| --- | --- | --- |
| Recruitment | 15 | Monthly newsletter with the total number of included patients |
| **Methods: Assignment of interventions (for controlled trials)** | | |
| Allocation: |  |  |
| Sequence generation | 16a | Centralized blocked randomization will be stratified on center and will be prepared by the clinical research Unit (URC-EST). |
| Allocation concealment mechanism | 16b | Investigators will be blinded for the randomization list and block size. |
| Implementation | 16c | Patients will be randomized after inclusion (STOP at 8 days or 7 DAYS PURSUIT) using the e-CRF (CleanWeb, Telemedecin Technologies, S.A.S) |
| Blinding (masking) | 17a | Open study |
|  | 17b | Not applicable |
| **Methods: Data collection, management, and analysis** | | |
| Data collection methods | 18a | Data will be collected in an electronic case report form (e-CRF), devised by the study coordinator in collaboration with URC-EST. Data will be completed by the investigators for each visit of follow up with the help of a Clinical Research Technician (CRT) of URC-Est for AP-HP centers and of each center for others centers. |
|  | 18b | Not applicable |
| Data management | 19 | Data entry will be carried out on electronic media via a web browser by investigators with the help of a TEC. Data entry will be monitored by CRA and will be checked for missing values and consistency by a datamanager. |
| Statistical methods | 20a | Outcomes:  Principal criteria analysis:  Since this is a non-inferiority study, analysis of principal criteria will be performed on per protocol population.  Morbi-mortality rate defined as mortality rate and recurrence during hospitalization in the ICU rate at D90 will be performed in each group.  Difference between groups will be performed and his two-sided confidence interval.  If the upper bound of the confidence interval is above the 10% of difference, the non-inferiority hypothesis of the short duration antibiotherapy group will be rejected. Non-inferiority will be tested by a Dunnet and Gent χ².  Secondary analysis in intent to treat (ITT) will be performed.  Secondary outcomes will be analyzed under superiority assumption. D30 and D90 mortality rates and acquisition of MDR during the hospitalization in the ICU will also be compared between groups by a Chi² test or a Fisher’s exact test, if the use conditions were not verified.  Duration of mechanical ventilation, duration of hospitalization in ICU, duration of exposure to antibiotics and number of extrapulmonary infections during hospitalization in the ICU will be compared between groups by Student tests or Mann-Whitney/Wilcoxon tests, if needed.  Types of extrapulmonary infections will be described.  The association between co-infection with yeast at diagnosis and P. aeruginosa recurrence will be studied by logistic regression adjusted on the randomization group. |
|  | 20b | Additional analysis:  The association between monotherapy or biotherapy and, mortality and P. aeruginosa recurrence will be studied by a logistic regression adjusted on the randomization group.  PCT levels at D0, D1, D3 and D7 will be described and their prognosis values on mortality and P. aeruginosa recurrence will be studied by logistic regression and sensibility and specificity analyses. |
|  | 20c | Population and missing data: Per protocol population will be defined as randomized patients without major protocol deviations such as non-respect of all selection criteria, clearly non-respect of antibiotic duration allocated (+/- 24 hours), missing data for the primary endpoint and major protocol deviation identified during a blinded data review before data base freezing. Missing data will not be replaced. |
| **Methods: Monitoring** | | |
| Data monitoring | 21a | A Data and Safety Monitoring Board (DSMB) was not requested either by the French ethics committee (Comité de Protection des Personnes Ile de France VI) or the French Drug Safety Agency (Agence Nationale de Sécurité du Médicament et des Produits de Santé. Consequently, no DSMB was instated for our trial. |
|  | 21b | Not applicable |
| Harms | 22 | The monitoring compliance with the treatment will be performed using the table of prescribed antibiotics present in the e-CRF. |
| Auditing | 23 | All data, documents and reports may be subject to regulatory audits and inspections. An audit can be carried out at any time by individuals appointed by the [sponsor](http://www.chusa.jussieu.fr/urcest/sous_cadre.php?fich=Lexique/new_index.php?isphp=0&fich=EC/legislation/DispositionslegislativesPromoteur.htm) and who are not associated with the research directors. |
| Ethics and dissemination | | |
| Research ethics approval | 24 | The study has received its approval from the French ethics committee (Comité de Protection des Personnes Ile de France VI), as well from the French Drug Safety Agency (Agence Nationale de Sécurité du Médicament et des Produits de Santé (EudraCT 2015-003102-17). |
| Protocol amendments | 25 | Not applicable at this stage. Amendements are submitted to the ethical committee and to the French Drug Safety Agency for aprobation. |
| Consent or assent | 26a | Local investigators will obtain informed consent from potential trial participants or authorised surrogates. |
|  | 26b | Not applicable |
| Confidentiality | 27 | Data will be collected in an electronic case report form (e-CRF), devised by the study coordinator in collaboration with URC-EST. Data will be completed by the investigators for each visit of follow up with the help of a Clinical Research Technician (CRT) of URC-Est for AP-HP centers and of each center for others centers. Source documents are defined as any original document or object that can prove the existence or accuracy of a piece of information or a fact recorded during the research. These documents will be kept for 15 years by the investigator or by the hospital in the case of a hospital medical file.  Source documents that will be kept for 15 years are completed and signed CRFs. |
| Declaration of interests | 28 | No conflict of interest directly related to the trial |
| Access to data | 29 | AP-HP, the academic sponsor of the study has the responsibility for the data access. |
| Ancillary and post-trial care | 30 | For the duration of the research, the Sponsor will take out an insurance policy covering the sponsor's own civil liability as well as the civil liability of all the doctors involved in carrying out the research. |
| Dissemination policy | 31a | Dissemination of the final research will be through targeted meeting and publications in the relevant journals. |
|  | 31b | No professional writer is planned. |
|  | 31c | There is currently no plan. |
| Appendices |  |  |
| Informed consent materials | 32 | Model consent form and other related documentation given to participants and authorised surrogates |
| Biological specimens | 33 | Not applicable |

*It is strongly recommended that this checklist be read in conjunction with the SPIRIT 2013 Explanation & Elaboration for important clarification on the items. Amendments to the protocol should be tracked and dated. The SPIRIT checklist is copyrighted by the SPIRIT Group under the Creative Commons “[Attribution-NonCommercial-NoDerivs 3.0 Unported](http://www.creativecommons.org/licenses/by-nc-nd/3.0/)” license.
